# Extraction of Phenolic Compounds and Terpenes from *Cannabis sativa* L. By-Products: From Conventional to Intensified Processes

**DOI:** 10.3390/antiox10060942

**Published:** 2021-06-10

**Authors:** Emilie Isidore, Hamza Karim, Irina Ioannou

**Affiliations:** URD Industrial Agro-Biotechnologies, CEBB, AgroParisTech, 51110 Pomacle, France; emilie.isidore@agroparistech.fr (E.I.); hamza.karim@cea.fr (H.K.)

**Keywords:** *Cannabis sativa* L., by-products, phenolic compounds, terpenes, biological activities, extraction processes, intensification

## Abstract

*Cannabis sativa* L. is a controversial crop due to its high tetrahydrocannabinol content varieties; however, the hemp varieties get an increased interest. This paper describes (i) the main categories of phenolic compounds (flavonoids, stilbenoids and lignans) and terpenes (monoterpenes and sesquiterpenes) from *C. sativa* by-products and their biological activities and (ii) the main extraction techniques for their recovery. It includes not only common techniques such as conventional solvent extraction, and hydrodistillation, but also intensification and emerging techniques such as ultrasound-assisted extraction or supercritical CO_2_ extraction. The effect of the operating conditions on the yield and composition of these categories of phenolic compounds and terpenes was discussed. A thorough investigation of innovative extraction techniques is indeed crucial for the extraction of phenolic compounds and terpenes from cannabis toward a sustainable industrial valorization of the whole plant.

## 1. Introduction

*Cannabis sativa* L. is one of the oldest annual crops originated from Asia. The *C. sativa* species is recognized to include many varieties classified according to their use [1]. Despite divergent opinion, *C. sativa* is usually divided into two subspecies categories based on their content in psychoactive molecules, mainly tetrahydrocannabinol (THC). Drug type category contains up to 20% THC on dry weight basis while non-drug types (hemp), must have a THC content lower than 0.2% in most European countries [2,3]. The drug type is well known for its recreational use. However, the therapeutic interest of cannabinoids resulted in a flexibility of the legislation and the legalisation of cannabinoids-based products in some countries [1,4]. Industrial hemp (non-drug type) is exploited for its fiber and oil [1]. Nowadays, climatic and economic conditions are pushing towards the use of plant raw materials to limit our dependency on petrochemical-derived products. As a result, renewed interest, due to its high fiber content, was shown in industrial hemp whose cultivation had been abandoned in the 19th century [5,6]. Industrial hemp has also been recognized for pharmaceutical uses as it contains secondary metabolites with potent bioactivities for human health [5]. The term cannabis refers to two main cultivars of *C. sativa*, hemp or drug types [1,7]. It is therefore used as an equivalent to the denomination *C. sativa*, when mentioned through this review. The cannabis industry generates many by-products either from unused plant parts (e.g., inflorescence, seeds) or residues from the defibration or extraction processes (e.g., dust, shives, and seed meal). The presence of many molecules with high added value in these different by-products suggests that a multi-valorization can be carried out on this plant.

Cannabis is a complex and rich plant that can produce more than 480 molecules [8]. Cannabinoids, phenolic compounds (PC) and terpenes are three, particularly interesting, chemical classes of secondary metabolites contained in *C. sativa*. Cannabinoids, which result from the condensation of PC and terpenes, are the most studied compounds [9]. Such an interest is mainly due to two well-known molecules, THC and CBD (cannabidiol), that exhibit numerous pharmacological properties such as analgesic, anti-inflammatory, and anti-cancer activities [10,11]. However, THC is also associated with soft drugs and exhibits several side effects, such as increased anxiety, cholinergic deficiencies, and immunosuppression [12]. A study led in 2019 reviewed all the extraction processes applied to recover cannabinoids and their derivatives, mostly from cannabis inflorescence as well as the different analytical techniques for their determination [13]. Ethanol showed the best solubility towards cannabinoids during hot maceration. Intensification processes (ultrasounds, microwaves) have been tested in an attempt to increase the efficiency of ethanolic extraction, showing no interest in comparison with dynamic maceration. Greener approaches such as supercritical fluid extraction or deep eutectic solvent extraction have led to interesting results but need further development.

Terpenes and phenolic compounds from *C. sativa* have been little exploited until now despite their beneficial biological activities and properties. Indeed, researchers have started to study their extraction and valorization only a few years ago. However, to our knowledge, no review on the extraction processes of these molecules from cannabis for their recovery have been published. Phenolic compounds are widely represented in this plant by three major classes: flavonoids, stilbenes and lignans [2,14]. It is well known in the literature that PC have numerous beneficial effects in the prevention of various diseases associated with oxidative stress mainly due to their antioxidant activity [15]. Thus, the extraction of these molecules without altering their activities is attracting an increasing amount of attention from researchers [16]. Terpenes are lipophilic hydrocarbon chains responsible for the odor and the flavor of cannabis. More than 100 of these molecules have been identified to date in *C. sativa*. The monoterpenes, sesquiterpenes and triterpenes represent the main terpenes. Numerous studies have shown that terpenes have many health properties such as anti-inflammatory, analgesic, anxiolytic, anti-bacterial, and anti-fungal activities [5]. Moreover, mono- and sesquiterpenes are the main components of the cannabis essential oil, so they can be used in cosmetics or as flavoring agents in the food industry [17]. It is also worth mentioning the researches contributing to understand the “entourage effect”, consisting in a synergy between cannabis metabolites, as reviewed by Nahler et al. (2019) [18]. Some terpenes and flavonoids, among other metabolites, are assumed to interact with cannabinoids and modify their properties, leading to the wide range of pharmacological effects observed depending on the different cannabis chemotypes [18].

Since the phenolic compounds and the terpenes present in the cannabis plant are of great interest for certain industries (e.g., pharmaceuticals, food) [2,14], the implementation of recovery strategies for these molecules seems relevant. Existing studies on the extraction of PC and terpenes are essentially focused on the characterization of the various cannabis by-products. To explore the issues associated with the extraction of PC and terpenes from cannabis by-products, it seems essential to report all the studies on the subject. Thus, the objective of this paper is to review the extraction techniques implemented for the recovery of PC and terpenes from cannabis as well as their operating conditions. The first part of this review will deal with the extraction of the phenolic compounds, the main PC identified in cannabis and their biological activities will be summarized, then the different extraction techniques will be presented. The second part, structured in the same way as the first, will focus on terpenes. A discussion of all the extraction techniques used for the three main classes of cannabis secondary metabolites (cannabinoids, PC and terpenes) will conclude this review.

## 2. Extraction of Phenolic Compounds from Cannabis

### 2.1. Phenolic Compounds Present in Cannabis and Their Biological Activities

**Figure 1:**
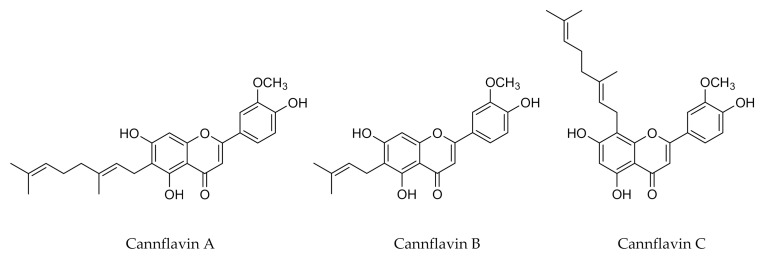
Specific flavonoids found in cannabis.

The *C. sativa* plant synthesizes PC (flavonoids, stilbenoids, and lignans) whose certain are unique [2,14]. At least twenty-six flavonoids have been identified in cannabis. These flavonoids can be glycosylated or aglycones. Some common flavonoids were found in cannabis (vitexin, isovitexin, apigenin, luteolin, kaempferol, orientin, and quercetin [7,8,19]), whereas others such as cannflavin A, B and C are unique to *C. sativa*. These are flavones associated with a geranyl group for cannflavin A and C, or prenyl group for cannflavin B (Figure 1).

The distribution of these components in cannabis varies highly between species and between the different organs of the plant. For instance, the orientin content is higher in the leaves than in the seeds, without significant differences between male and female plants and cannabis varieties, whereas the presence of quercetin is higher in male flowers with important variation between male flowers of hemp and drug types [20]. These differences are crucial, knowing that *C. sativa* can have female and male flowers on the same plant (monoecious cultivars) or on separate plants (dioecious cultivars) [2,3,21]. As a result, cannabis flavonoids have been isolated and detected in different parts of the plant such as flowers, leaves, twigs, and pollen [22,23].

Remarkable biological activities of cannflavins have been reported. In vivo tests have demonstrated anti-inflammatory activity as well as anti-cancer and anti-leishmanial activities. These components can relieve pain up to thirty times more than aspirin [24,25,26,27,28,29]. Flavonoids, being known as great antioxidant agents, appear to be responsible of the significant antioxidant potential of hemp oil [30].

**Figure 2:**
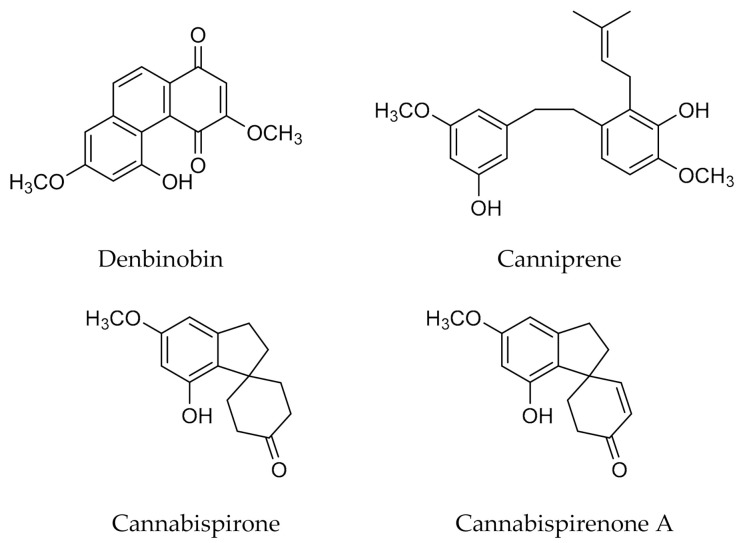
Examples of stilbenoids from *Cannabis sativa* L.

Cannabis stilbenoids can be divided into three main types based on their structure: phenanthrenes, dihydrostilbenes, and spiroindans (Figure 2). These components have been isolated from the cannabis stem, leaves and flower heads [31,32,33,34,35]. Despite their association with disease resistance and human health [36,37], only a few cannabis stilbenoids have been described in the literature with properties that may be beneficial to human health. However, denbinobin, one of the best characterized stilbenoids in cannabis, has been shown to have significant pro-oxidant and pro-apoptotic activity against human leukemia cell lines [38]. Among the dihydrostilbenes, canniprene has attracted some attention as this compound unique to cannabis showed an anti-inflammatory activity. Cannabispirone and cannabispirenone A, the most recurrent spiroindans, have also been studied and anti-inflammatory and anti-cancer activities have been reported. Furthermore, a competition in the formation of stilbenoids and flavonoids is suggested in *C. sativa* metabolism [2,29,34,36].

**Figure 3:**
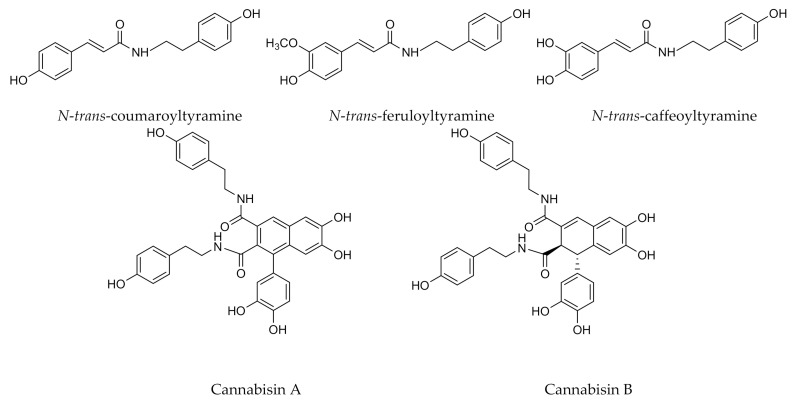
Major lignans present in cannabis.

The lignans isolated from *C. sativa* belong to two main groups: phenolic amides and lignanamides (Figure 3) [7,39]. Phenolic amides of *C. sativa* result from the reaction of a phenolic acid and an amine such as tyramine or octopamine [40]. Three major phenolic amides have been recovered from cannabis. These are, *N-trans*-coumaroyltyramine detected in the roots of *C. sativa* [41], *N-trans*-feruloyltyramine and *N-trans*-caffeoyltyramine isolated from cannabis seeds [2]. *N-trans*-caffeoyloctopamine and *N-trans*-coumaroyloctopamine have also been identified recently [40]. Regarding lignanamides, the cannabisin molecules and grossamide have been extracted from cannabis. Lignanamides result from the oxidative coupling of phenolic amides, they also occur in *Solanaceae* and *Papaveraceae* families [40,42]. Lignans are molecules of great interest where they can find applications in the pharmaceutical sector for their various properties. For example, *N-trans*-caffeoyltyramine and cannabisin A demonstrated a strong antioxidant activity, while anti-inflammatory properties have been reported for grossamide and cannabisin F; cannabisin B showed a cytotoxic activity as well [39,42,43].

The content of the different categories of PC varies according to varieties, geographical locations and plant organs. Flavonoids and stilbenoids are found mainly in the aerial parts (leaves, flowers, stems) while lignans are located in the roots, seeds and fruits [2,7,20]. Interestingly, PC were quantified in cannabis floral water at a content of 28.04 mg gallic acid equivalent (GAE)/g extract, including 14 w% of flavonoids and 6 w% of phenolic acids [44]. Ferulic and coumaric acids are also present in hemp fiber, hurds and dust [45,46,47]. Moreover, an evolution of PC is noticeable during the plant growth, where their content decreases during the growth due to their condensation with terpenes to form cannabinoids [9].

### 2.2. The Extraction Processes of PC from Cannabis

Liquid-solid extraction techniques are used for the recovery of PC from plant biomass, conventional solvent extraction (CSE) being predominant. However, some authors used intensification techniques such as ultrasounds, microwaves or the use of high pressure to improve the process efficiency.

#### 2.2.1. Conventional Solvent Extraction

The extraction of PC from cannabis in a conventional manner has been explored in several studies. Table 1 presents the operating conditions and the yields of the PC obtained for each study.

Based on the articles identified, it can be said that most of the work that has been done using conventional solvent extraction has dealt with the characterization of PC in cannabis. Thus, studies essentially focused on determining the recovery potential of these molecules from cannabis by-products such as seed meals or leaves. The majority of the extractions were conducted at room temperature [48,49,50,51,53,54], though an increase in temperature leads to a higher PC recovery [54]. Only one study optimized the extraction temperature carried out with a new eco-friendly solvent, an aqueous solution of 2-hydroxypropyl-β-cyclodextrin (CD) [52]. Cyclodextrines are hydrotropic agents which increase the solubility of hydrophobic solutes in water [55]. There are other examples of such substancies, the hydrotropic solvents are their aqueous solutions. This eco-friendly solvent has already been successfully used for the extraction of polyphenol from Vine shoots [56]. Extraction time and solvent/solid ratio vary between studies. Optimization studies to find the optimum operating conditions are lacking. Mkpenie et al. (2012) [51] investigated the influence of the extraction time during maceration at room temperature. They found that the recovery of PC increases by 60% between 2 and 8 h of extraction. This trend is also noticeable at higher temperatures, where the extraction yield is increased by almost 2-fold between 2 and 6 h of soxhlet extraction while the TPC remain constant in the extract [54]. The type and concentration of solvent have been relatively more studied than the other parameters, but more work is still needed.

It should be noted that the chemical profile of cannabis varies depending on the type of cultivar and the growing conditions. This is clear from the results obtained by Lesma et al. (2014) [48]. The phenol and flavonoid contents of the seeds of three industrial hemp cultivars, grown in two sites were determined after extracting the defatted plant material using a mixture of MeOH: water (80:20 *v*/*v*). The TPC ranged between 67.0 and 80.4 mg GAE/g of extract depending on the cultivar type, for a given culture location. Moreover, when the TPC of the same cultivar type is compared between the two sites, a significant difference in the final yield was noticed. For instance, TPC of the *Futura* type increased about twice when grown in the *Cavriana* region. Similar variations within varieties and cultivation sites are observed with flavonoid contents, and appear with stilbenoids content as well [34,48]. Therefore, the results from different studies cannot be compared with each other and this is the case with the other extraction techniques.

Regarding hemp seed meal, a study conducted in 2014 compared the influence of methanol (80, 100%), acetone (80%, 100%), ethanol (100%) and a mixture of methanol: acetone: water (MAW 35:35:30% *v*/*v*/*v*) on the final yield of PC [50]. In this study, the extraction was held at room temperature for a period of 1 h. In the end, the results demonstrated that the MAW mixture gives the most interesting results, followed by 80% acetone and 80% MeOH then pure acetone and MeOH. These findings are consistent with the results of Mkpenie et al. (2012) [51]. During this work, the performances of absolute methanol, acetone, and their 50% aqueous mixture were compared using hemp leaves as plant material. Their results also demonstrated that acetone was more suitable than methanol for an extraction time of 2 h. However, by increasing the extraction time to 8 and 18 h, pure MeOH results in higher yields (0.89 mg GAE/g cannabis), giving 2 times more phenolic content than absolute acetone (0.416 mg GAE/g cannabis). Methanol was also found to be more efficient among pure solvent during long maceration of *C. sativa* leaves [53].

Mourtzinos et al. (2018) [52] used an innovative approach by extracting ground hemp meal with an aqueous solution of 2-hydroxypropyl-β-cyclodextrin (CD). Optimal conditions gave a higher phenolic yield in comparison with methanol, ethanol and water. CD have the property to form inclusion complexes with PC resulting in increased contents.

The effect of different concentrations of ethanol in water (30%, 50%, 70%, and 90%) on the extraction yield, TPC and TFC of aerial parts of hemp has been examined by Dirinić et al. (2018) [49]. The results showed that 50% EtOH is the best solvent for PC recovery, with an extraction yield of 15.68 w%, a TPC of 17.05 mg GAE/g dry weight of plant material (DW), and a TFC of 11.20 mg catechin equivalent (CE)/g. However, this optimal concentration of EtOH in water could not be compared to the performance of other solvents such as acetone and MeOH.

Cannabis roots have been briefly explored, where 0.01% DW of *N*-*trans*-coumaroyltyramine were isolated from an ethanolic extract [41]. Likewise, stilbenoids have been extracted with polar [31,34,35] and apolar [33] solvents without any process optimization. A deeper investigation of the extraction might possibly lead to a better yield for these compounds.

In most studies, the antioxidant activity of the extracts was evaluated using mainly in vitro assays (Table 1). In general, PC where correlated to an antioxidant activity [3,50,51,52]. As a consequence, the extraction parameters had the same effect on the extraction of PC and the resulting antioxidants capacity of the extract. In addition, the extracts have been tested on diverse cell lines, bacteria and fungi, showing antioxidant activiy against human erythrocytes [3], antimicrobial activity [3,51] and anti-inflammatory activity [34].

The small number of studies identified demonstrates the need of additional work to assess the efficiency of CSE. Kinetic studies would enable a better definition of the minimum extraction time to reach equilibrium as well as the diffusion rate of the molecules into the solvent. The most used solvents for the extraction are acetone, MeOH, EtOH and their aqueous mixtures [3,48,49,50,51,52]. The latter appear to be the most efficient for extracting phenolic compounds from hemp. However, optimization studies should be carried out to investigate the effects of the nature of the solvent, its concentration and the extraction temperature and their interactions on the yield of PC. The sample preparation also appears to influence the contents released. Differences in yield appear depending on the state of the material (fresh or dried) and the particle size. Considering the need to find sustainable processes, it also seems important to explore eco-friendly solvent extraction.

#### 2.2.2. Ultrasound Assisted Extraction (UAE)

UAE is an extraction technique using ultrasound waves, mechanical vibrations that pass through the extraction medium. The waves create acoustic cavitation through the generation of cycles of expansion and compression, inducing the formation of bubbles which expand and collapse. These effects provoke the destruction of cell walls, thereby releasing the cell contents [57,58].

Table 2 summarizes the different operating conditions used and the results obtained in the different studies using UAE process for the extraction of PC from cannabis.

Most UAE is done using an ultrasonic bath with methanol or its aqueous mixture as a solvent. These two solvents are often used for the extraction of antioxidants from plants [59]. The influence of the nature of the solvent on the extraction of PC using ultrasounds has been carried out on defatted seed and meal [43,60]. The solvents used are water, MeOH, EtOH and acetone at various concentrations. Pure solvents and water led to a lower extraction of PC in comparison with a mixture of aqueous solvents, emphasizing the importance of the polarity of the solvent for the extraction. The optimum yield is obtained for an aqueous mixture of 75% or 80% acetone.

**Table 2:** Studies using ultrasound assisted extraction technique for the recovery of phenolic compounds.

Plant Variety	Plant Organ	Solvent	Operating Conditions	Best PC Results	Best Biological Activities	Ref.
Kompoti, Tiborszallasi, Antal, Carmagnola Cs	Inflorescence	MeOH	150 mL/g, 4 °C, 30 min	Total identified polyphenols up to 743.5 mg bioactives/kg sample (Carmagnola Cs) Up to 36.1 mg *N*-*trans*-caffeoyltyramine/kg sample (Carmagnola Cs) Up to 2.12 mg lignanamides/kg sample (Carmagnola Cs) Up to 72.9 mg Cannflavin A/kg sample (Tiborszallasi) Up to 98.8 mg Cannflavin B/kg sample (Antal)	DPPH up to 63.6 mmol trolox/kg (Carmagnola Cs)	[14]
Bialobrzeskie, Felina 32, Tygra, Futura 75, Santhica 27, Fedora 17, Finola	Defatted seeds	MeOH 80%	65 °C, 30 min	TPC up to 7.8 mg GAE/g seeds (Futura 75) Major phenolic amide: *N*-*trans*-caffeoyltyramine up to 0.8 mg GAE/g seeds (Futura 75) Major lignanamide: cannabisin A up to 1.6 mg GAE/g seeds (Futura 75)	ABTS up to 10.7 mg TE/g (Futura 75) FRAP up to 8.1 mg TE/g (Futura 75)	[21]
Ermes, Santhica 27, Ermo	Inflorescence	MeOH	100 mL/g, 50 °C, 1 h	Cannflavin A up to 0.024% DW (Ermo)	Nd	[61]
Fedora	Seeds	MeOH 80%	10 mL/g, 4 °C, 30 min	0.767 mg GAE/g seeds	51.5% DPPH inhibition, corresponding to 5.2 mmol trolox equivalent	[62]
	Defatted seed flour			0.744 mg GAE/g flour	46.8% DPPH inhibition, corresponding to 4.7 mmol trolox equivalent	
	Oil			0.021 mg GAE/g oil	8.2% DPPH inhibition, corresponding to 0.36 mmol trolox equivalent	
Alyssa, Anka, CanMa, CFX 1, CFX 2, CRS 1, Delores, Finola	Seeds	MeOH 90%	9 mL/g, cold room, 30 min	TPC up to 51.6 mg GAE/g DW (Anka).	Nd	[63]
Fedora	Seeds	MeOH 80%	10 mL/g, 30 min	0.922 mg GAE/g seeds	74% DPPH inhibition	[64]
	Defatted seed flour			1.709 mg GAE/g flour	67% DPPH inhibition	
	Oil			0.192 mg GAE/g oil	22% DPPH inhibition	
Futura 75, Kc virtus, Carmagnola Cs, Villanova	Inflorescence	Water	50 mL/g, 60 °C, 10 min	TPC up to 8.1 mg GAE/g extract (Futura 75 and Villanova) TFC up to 6.3 mg RE/g extract (Kc virtus)	Antioxidant and anti-inflammatory activiy of extracts (assays on cell lines and rats) Anti-microbial activiy of Futura 75 extracts	[65]
Helena	Seed meal	MeOH 80%	80 mL/g, 10 min	*N-trans*-caffeoyltyramine up to 0.287 mg/g DW (>350 µm fraction) Cannabisin B up to 0.153 mg/g DW (>350 µm fraction) *p*-hydroxybenzoic acid up to 0.129 mg/g DW (>180 µm fraction) Catechin up to 0.744 mg/g DW (>180 µm fraction)	DPPH: IC50 up to 5.29 mg/mL (>350 µm fraction)	[66]
Bama, Yunma	Defatted kernels	MeOH, EtOH and acetone at 50% 75% 100%	10 mL/g, 30 min	Best solvent: acetone 50% TPC up to 16.1 mg GAE/g extract in Bama kernels	DPPH: IC50 up to 0.58 mg/mL (Yunma kernels) ABTS: IC50 up to 0.053 mg/mL (Yunma kernels)	[43]
	Defatted hulls			Best solvent: acetone 75% TPC up to 139.3 mg GAE/g extract in Bama hulls	DPPH: IC50 up to 0.09 mg/mL (Bama hulls) ABTS: IC50 up to 0.012 mg/mL (Bama hulls)	
Hungary	Mix of leaves, flowers and seed husks	MeOH, 20, 50, 80%	20 mL/g, 90, 120, 150 W, 5, 10, 15 min	Optimal conditions: 15 min, 130 W, 80% MeOH Obtained values: TPC 312.452 mg GAE/g DW, TFC 32.254 mg QE/g DW	In optimum conditions, FRAP: 17.84 mM ascorbic acid equivalent/g DW	[59]
Nd	Seed cake	MAW (35:35:30)	5–20 mL/g, 40–70 °C, 20, 30, 40 min, 200 W	Optimum extraction conditions: L/S = 10, 20 min, 70 °C TPC 15.42 mg GAE/g FW TFC 0.23 mg LUE/g FW	30.94% DPPH inhibition in optimum conditions FRAP: 16.74 µmol Fe(II)/g fresh weight in optimum conditions	[67]
Nd	Hempseed cake	MeOH 70%, Acetone 80%, MeOH 70%:Acetone 70% (1:1 *v*/*v*)	9 mL/g, 1 min	Best solvent: acetone 80% (TPC = 4.29 mg GAE/g DW) *N-trans*-caffeoyltyramine = 0.91 mg CatE/g DW Cannabisin B or isomers = 1.20 mg CatE/g DW	Nd	[60]
Futura 75	Inflorescence	EtOH	5 mL/g, 15 min	TPC 110.30 mg GAE/g extract Gallic acid 35.10 µg/g extract Caffeic acid 81.81 µg/g extract Syringic acid 57.28 µg/g extract Rosmarinic acid 514.33 µg/g extract Luteolin 1384.09 µg/g extract	FRAP: 77.22 mg TE/g extract DPPH: 45.04 mg TE/g extract ABTS: 381.26 mg TE/g extract	[54]
Skunk, Fourway, Kompolti, Fasamo	Fruits	MeOH 50%:CHCl_3_ (1:1)	80 mL/g, 10 min	TFC up to 0.06 mg/100 mg DW	Nd	[20]
	Seedling			TFC up to 1.67 mg/100 mg DW Major flavonoids: vitexin, luteolin and apigenin		
	Bracts			TFC up to 2.18 mg/100 mg DW		
	Leaves			TFC up to 2.54 mg/100 mg DW Major flavonoids: orientin, vitexin, isovitexin, luteolin, kaempferol and apigenin		
	Flowers			TFC up to 1.56 mg/100 mg DW Major flavonoids: quercetin, luteolin, kaempferol in male flowers		

ABTS: 2,2′-azino-bis (3-ethylbenzothiazoline-6-sulphonic acid). CatE: catechin equivalent. DPPH: 2,2-diphenyl-1-picrylhydrazyl or 1,1-diphenyl-2-picrylhydrazyl. DW: dry weight. FRAP: ferric reducing antioxidant power. FW: fresh weight. GAE: gallic acid equivalent. IC50: concentration of extract allowing 50% of inhibition/ion reduction. L/S: liquid-solid ratio. LUE: luteolin equivalent. MAW: methanol, acetone, water. Nd: not defined. QE: quercetin equivalent. RE: rutin equivalent. TE: trolox equivalent.

Only a few authors have worked on the optimization of UAE for the recovery of phenolic compounds. Agarwal et al. (2018) used UAE to extract bioactive compounds from *C. sativa* [59]. They studied the influence of three independent parameters: time, input power, and MeOH concentration. The extraction time and the solvent concentration has a significant influence on the PC yield. However, the combined effect of time and power has a negative influence on TPC and TFC. Indeed, an excessive increase in the sonication time does not promote the extraction but the degradation of the target molecules due to the rise in temperature. Compared to conventional solvent extraction, UAE led to a 2-fold increase in TPC and TFC, in a shorter time. Teh et Birch (2018) [67] optimized the ultrasonic treatment of hemp seed meals using a MeOH:acetone:water (35:35:30, *v*/*v*/*v*) mixture. Different liquid/solid ratios (5–20), extraction temperatures (40–70 °C), and extraction times (20–40 min) were studied. The temperature of 70 °C is the optimum temperature for obtaining a maximum TPC while the maximum TFC is obtained at 50 °C. As found previously, an extraction time exceeding 20 min leads to a decrease in the yield of PC.

Ultrasound assisted extractions were carried out on different parts of hemp, different varieties cultivated in different places leading to obtaining multiple phenolic contents and profiles. Thus, the comparison of data from the literature should be done with caution. Izzo et al. (2020) [14] evaluated the phenolic fraction of the inflorescence of four different hemp cultivars. The TPC vary between 10.510 and 48.875 mg GAE/g depending on the cultivar [14] while Ferrante et al. (2019) [65] obtained between 4.7 and 8.1 mg GAE/g of extract, and Palmieri et al. (2020) [54] found 110.30 mg GAE/g of extract. These results cannot be compared because the PC contents are expressed in different matrix unit. However, the second result can be considered as a low value because the extraction was performed with water, which is not a preferred solvent for the PC extraction according to Chen et al. [43]. Among the tested aerial parts, the highest flavonoids content are detected in cannabis leaves and bracts [20].

Flavonoids were found to be the major class of PC in inflorescences, representing approximately 80 w% of the components identified. Flavones, more particularly cannflavins A and B are part of the most abundant flavonoids. Their average concentration varies, respectively, between 61.8 and 84.5 mg/kg of fresh weight, which is equivalent to a rate ten to one hundred times higher compared to other parts of the plant [14]. Another study found cannflavin A up to 240 mg/kg and estimated a production yield between 0.1 and 0.5 kg·ha^−1^ [61]. Among the other flavonoids represented, the flavonols quercetin-3-glucoside and the catechin were found at significant concentrations. Phenolic acids account for 18.6 w% to 29.7 w% of the compounds identified, including chlorogenic acid, caffeic acid, *p*-coumaric acid and ferulic acid. Lignanamides (cannabisins A, B, C) and the phenol amide *N*-*trans*-caffeoyltyramine are also represented with an average value of 1.0 mg/kg for cannabisin A and 23.7 mg/kg for *N*-*trans*-caffeoyltyramine. Cannabisins B and C are present in lower quantities [14].

Hemps seeds, oil, and meals have also been characterized through UAE. The TPC of hemp seeds varies between 0.767 and 51.6 mg GAE/g depending on cultivar type and extraction temperature (4–65 °C) [21,62,63,64]. The most abundant PC detected in seeds belong to the lignan class; particularly *N-trans*-caffeoyltyramine and cannabisin A present at concentrations ranging up to 0.832 mg *trans*-cinnamic acid equivalent (CAE)/g and 1.591 mg CAE/g of seeds, respectively. Minor phenolic acids (protocatechuic acid, *p*-hydroxybenzoic acid, cinnamic acid, vanillic acid, *p*-coumaric acid, and ferulic acid) were also measured [21]. Moccia et al. (2020) [64] and Siano et al. (2019) [62] studied the PC contents in hemp seeds, oil and flour (ground seed meals). Seeds and flour have a similar profile while seed oil is characterized by a low TPC (0.021–0.192 mg GAE/g) with a profile characterized by phenolic acids, flavonoids and cannabinoids. Indeed, most phenolic compounds are not transferred into the oil during its extraction [62,64]. This is also the case for several other plants where polar antioxidant such as phenolic acids, flavonoids and lignans remain in the seed meal [68] which provide a potential valorization of these by-products. Pojić et al. (2014) [66] and Chen et al. (2012) [43] described the chemical composition of hemp seed meals according to meal fractions, corresponding to hulls and cotyledons. Their findings indicate that two strongly antioxidant compounds *N-trans*-caffeoyltyramine (0.267–0.287 mg/g) and cannabisin B (0.117–0.153 mg/g) were found in high proportions in the hull fractions. Regarding the cotyledon fractions, catechin (0.313–0.744 mg/g) and hydroxybenzoic acid (0.124–0.129 mg/g) were found and quantified. These results indicate that the separation of hemp seed meal into different fractions could be used to facilitate the recovery of the target compounds.

The antioxidant capacity of ultrasound assisted extracts of PC from *C. sativa* where evaluated (Table 2). Like in CSE, a correlation between PC and the antioxidant activity of the extracts was highlighted [21,43,67]. In particular, the phenol amide *N-trans*-caffeoyltyramine and the lignanamides cannabisins A and B showed a strong protection against DPPH and ABTS oxidation, and ferric reduction [21,43].

It is known from CSE that liquid/solid ratio, solvent, temperature and time have an influence on PC extraction. The optimal UAE temperature and liquid/solid ratio was claimed to be 70 °C and 10 mL/g, respectively [67], when these parameters were not optimized in CSE. The intensification technique is then difficult to compare to CSE without a proper definition of the optimum conditions in both techniques. Among the tested extraction solvents, aqueous acetone (75–80%) is the optimal solvent in both cases. Like in CSE, the exploration of eco-friendly solvents is lacking in the reviewed articles. Particular attention shall be given to ultrasonic time and its interaction with ultrasonic power according to the cited articles. In any case all studies agreed on the time saving of UAE in comparison with CSE.

#### 2.2.3. Other Extraction Techniques

Microwave-assisted extraction (MAE), pressurized liquid extraction (PLE), extrusion and rapid solid–liquid dynamic extraction (RSLDE) have also been used to recover PC from cannabis. Microwaves are electromagnetic radiations that can penetrate plant biomass and interact with polar molecules. The water contained in the biomass absorbs the microwave energy and induces rapid heating of the cells, causing their disruption and the release of the desired components [58]. MAE is an excellent green extraction technology and is particularly appropriate for the recovery of polar molecules such as phenolic compounds from plant materials [69]. However, regarding the extraction of phenolic compounds from cannabis, only three articles were identified. Drinić et al. (2020) [70] and Matešić et al. (2020) [71] focused on the optimization of MAE operating conditions during PC extraction from hemp. Both studies worked with ethanol:water at different ratios. It appears that the L/S ratio, and ethanol concentration had a significant influence on PC recovery. Depending on the targeted PC, Drinić and co-authors [70] noted different effect of each extraction parameter. For example, the quadratic term of ethanol concentration had a negative impact on TPC, meaning that high ethanol concentrations lead to a lower TPC yield; the opposite effect was observed for TFC recovery. The extraction time had a negative impact on TPC but had no significant effect on TFC [70]. The temperature and its interaction with microwave power were found to have a significant effect on TPC as well [71]. The optimum operating conditions for TPC extraction gave the highest DPPH inhibition and reducing power [70]. In both cases, the MAE is faster compared to maceration. The optimum MAE times are claimed to last 5 to 10 min, while minimum 40 min is needed in CSE. Teh et al. (2014) [72] used and optimized microwave technology as a pre-treatment before UAE. The operating conditions tested were water as solvent with liquid/solid ratios between 4 and 6 mL/g, a microwave input power equal to 440, 700 or 1100 W, and times of extraction ranging from 1 to 5 min. The optimum conditions obtained by applying the response surface methodology are a L/S ratio of 6 mL/g, an input power of 700 W, and an extraction time of 5 min. Extraction by ultrasounds of the biomass pretreated by microwaves yielded respective TPC and TFC contents of 13.31 mg GAE/g FW and 0.19 mg LUE/g FW. The resulting extract showed 27.52% of DPPH inhibition and a reducing power of 15.64 μmol Fe(II)/g FW. Similarly to ultrasounds, the authors underlined the influence of the microwave power, in particular the appearance of degradations due to an excessive temperature in the material generated by microwave power and its interaction with time.

Pressurized liquid extraction (PLE) is a technique used to perform extractions from solid and semi-solid samples under pressure. Accelerated solvent extraction (ASE) is an equivalent technique, the equipment named identically can operate at room temperature as well as temperatures above the boiling point of solvents, using pressure to keep the solvent in its liquid state [73]. Like MAE, only a few studies have used PLE for the recovery of PC from cannabis. Kitrytė et al. (2018) [74] used this technology to develop and optimize a multi-step biorefining process for the isolation of cannabinoid and antioxidant fractions from hemp aerial parts. Their biorefining approach included three consecutive processes, a supercritical CO_2_ extraction for the isolation of cannabinoids, a PLE for the extraction of PC, and a final step with enzyme assisted extraction (EAE) to break the cell wall and release its interesting compounds. During the PLE extraction, acetone was applied during optimized conditions of extraction (100 °C, 45 min), followed by an EtOH aqueous mixture in the same conditions. The acetone and ethanolic fractions resulted in TPC contents of 5.02 mg GAE/g DW and 23.52 mg GAE/g DW of initial plant material, respectively. In accordance with the cited results, most identified flavonoids were in the ethanolic fraction. Temperature was an important parameter: an increase to 100 °C enables better extraction of PC while extraction at 130 °C leads to a color change and potential degradation of PC. In addition, the ethanolic fraction showed the highest antioxidant capacity. A strong correlation was found between PC and the antioxidant activity of the extract, in particular using ABTS assay. ASE is usually considered to be an interesting technique to replace CSE because a better extraction efficiency is achieved using less solvent in a shorter time [73] as in the work of Bassil (2015) [46]. The focused was on the extraction of coumaric and ferulic acids from hemp hurds and dust. ASE yielded 1.5 times more total phenolic content in 30 min than CSE in 24 h. However, ASE was equivalent to CSE in the case of dust.

## 3. Extraction of Terpenes from Cannabis

Before exploring the different extraction processes applied for the recovery of terpenes from cannabis, the main terpenes found in cannabis are presented.

### 3.1. The Terpenes Present in Cannabis sativa L. and Their Biological Activities

**Figure 4:**
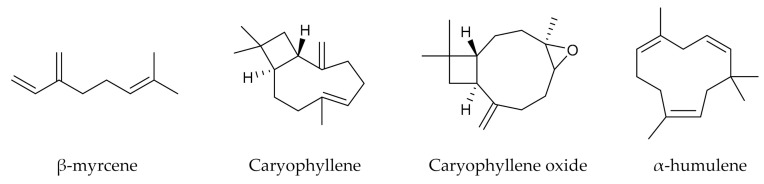
Notable terpenes of cannabis essential oil.

Terpenes are volatile compounds mostly present in the inflorescences of cannabis, more precisely in glandular trichomes [75]. The predominant terpenes of *C. sativa* are represented in Figure 4.

The terpenes are among the main compounds of the essential oil (EO) of cannabis [76]. Cannabis’ EO is a liquid with a yellow color more or less pronounced, composed of mono- and sesquiterpenes but also of other molecules such as terpene alcohols and cannabinoids [75,77,78,79,80]. Diverse biological activities have been associated with *C. sativa* EO including insecticidal, nematicide, antimicrobial, fungicidal, anti-leishmanial, antioxidant, anti-acetylcholinesterase or neuroactive activities [81,82,83,84,85]. The terpenes present in cannabis EO are responsible for the smell of the plant [7,81]. Based on the studied articles, the yield of cannabis EO can reach up to 0.55% of dry matter [82], the most abundant monoterpene found is often β-myrcene. This compound described in Figure 4 have demonstrated anti-inflammatory, analgesic, and anxiolytic activities and is used as a flavoring agent, solvent or additive of lubricating oils. Caryophyllene, also used as flavoring agent, is the most abundant sesquiterpene in many cultivars. Other sesquiterpenes present in significant quantities such as humulene and caryophyllene oxide can be identified in the EO of cannabis, the latter is the degradation product of caryophyllene (caused by oxidation). These compounds have shown an important anti-inflammatory activity as well as anticancer, analgesic and antipyretic properties, mainly for caryophyllene and caryophyllene oxide [5,75,81,86,87].

Since terpenes constitute the major components of cannabis EO, studies of EO are often linked to terpenes. Cannabis EO yield and the terpene contents vary significantly according to the varieties, and between monoecious and dioecious varieties [88]. Moreover, sesquiterpenes are the highest in the earlier harvest time while monoterpenes appear to increase in comparison to sesquiterpenes in the late harvest time, especially when the crop is subject to a water stress [75,86,89,90]. A difference in terpene contents in the inflorescence according to their position along the stem was noticed. Higher yields are found in the upper parts [79]. A loss of terpenes and oxidative reactions of sesquiterpenes are induced by the process of drying, forming caryophyllene oxide and humulene epoxide II from caryophyllene and humulene, respectively [87,91]. Interestingly, two terpenes (carvone and dihydrocarvone) have also been isolated from an hexane fraction of cannabis roots [92].

Apart from mono- and sesquiterpenes present in the EO, triterpenes have also been recovered from cannabis roots, stem and leaves. The highest content of triterpenoids was found in roots (up to 0.24 w%), where friedelin is the major compound identified at a content up to 0.135 w% [93]. Various other triterpenoids including epifriedelin and β-amyrin have also been detected in smaller amounts [41,45,93]. Friedelin have shown various biological activities including antiulcer, antidiabetic and cytotoxic effects [94,95,96] while β-amyrin have been associated with anti-microbial, anti-fungal properties and a potential anti-inflammatory activity [97]. To our knowledge, no biological activities have been reported for epifriedelin.

### 3.2. The Extraction of Terpenes from Cannabis

As seen in the previous section the recovery of terpenes consists of extracting EO. Most studies reported here involve hydrodistillation for the extraction of terpenes from *C. sativa*. This is one of the traditional methods for the recovery of EO from plant biomass. Some authors used CSE while others explored innovative methods, in some cases attempting to intensify or partition the extraction. As mentioned above, biomass conditioning has a significant effect on the EO yield and composition. Drying and storing the plant lead to a significant decrease in the percentage of EO, as observed in several studies [83,87,91]. The two processes cause the evaporation of high vapor pressure components. In particular, EO composition is characterized by a higher loss of the most volatile components, monoterpenes, and therefore an increased percentage of sesquiterpenes [83,87,91]. A further decrease in the EO yield occurs when the drying temperature is increased [98].

#### 3.2.1. Recovery by Steam and Hydrodistillation

Hydrodistillation (HD) and steam distillation (SD) are among the most popular methods used to extract EO from plant sources. These methods were developed to lower the boiling point molecules using the water vapor pressure [99]. The water steam penetrates the biomass and dissolves the volatile compounds. Solvent and solutes are then condensed that causes their separation, the upper phase of the liquid being the essential oil. The slight difference in the methods is that the steam is directly brought to the plant material in SD while the material is initially soaked in water and heated to its boiling point in the case of HD [100].

It appears that the type of distillation influences the selectivity of the process towards terpenes. The recent study of Fiorini et al. (2019) [87] compared the chemical composition of essential oil from Felina cultivar obtained by SD and HD. They noticed that with SD a greater content of monoterpenes (54%) is obtained compared to sesquiterpenes (44.2%). On the contrary, HD led to an abundance of sesquiterpenes (48.5%) compared to monoterpenes (43.9%). Caryophyllene remains the most abundant sesquiterpene in the EO regardless the process used. Another observation is that cannabinoid content increased significantly when using HD (6.1% instead of 0.1% with SD). This can be explained by an increase in the decarboxylation reaction with the HD process, which leads to their alcoholic form that possess a higher volatility. HD is considered as a better process due to the higher percentage of bioactive compounds (caryophyllene and cannabidiol) extracted. This is because, for SD process, a lower pressure is involved, and the steam does not penetrate uniformly the plant material. Therefore, more time is needed to extract components with higher boiling points [80]. Different temperatures were tested during SD and HD of cannabis inflorescences. The optimum temperatures are 110 °C and 130 °C for HD and SD, respectively. The EO yield increases with temperature in the case of SD, where higher temperatures are needed probably due to slower extraction kinetics [80]. Extraction partition during HD, combined with the effect of grinding, have been explored [101]. Interestingly, monoterpenes are extracted in the earliest minutes, thus a high-monoterpene content EO is recovered in the range of 0–10 min while the sesquiterpene-rich fraction is collected after 80 min. Ground material leads to an additional increase in monoterpene content but lowers the sesquiterpene fraction. It is worth noting that the cannabinoids are extracted in the last minutes, shorten distillation times will provide a cannabinoid-free EO.

#### 3.2.2. Conventional Solvent Extraction

The main objective of studies dealing with the extraction of terpenes from cannabis using CSE is the identification of these compounds. Cannabis EO are well extracted with both polar [78,79] and nonpolar solvent [75,79,89], however Namdar et al. (2018) [79] determined that a mixture of polar and nonpolar solvents results in the most efficient extraction of terpenes and cannabinoids from inflorescences. Indeed, the authors showed that the solvent mixture hexane:EtOH (7:3 *v*/*v*) is the most efficient for the extraction in comparison with pure hexane and ethanol. The use of polar solvent benefits to the cannabinoids yield [79]. Although the operating conditions are not detailed, L/S ratios vary in the range of 5–50 mL/g, the extractions last up to 1 h and the application of heat does not seem necessary [75,78,79,89]. Besides, high temperature during extended extraction time may cause a loss of volatile compounds and thus lower the EO yield.

Triterpenes have been extracted from cannabis roots and stem bark through CSE, using polar solvents. On one hand friedelin and epifriedelin were isolated from cannabis roots after an ethanolic extraction [41]. On another hand, cannabis stem bark were shown to contain small quantities of various triterpenes, identified from an acetone soxhlet extract [45]. No optimization of the extraction was performed for the recovery of terpenes nor triterpenes of EO from cannabis roots, although a deeper knowledge of CSE of triterpenes in particular may provide a better view for a potential valorization of these compounds.

#### 3.2.3. Supercritical Fluid Extraction

Supercritical Fluid Extraction (SFE) is an environmentally friendly extraction technique that uses a fluid in its supercritical state for the extraction process. CO_2_ is by far the most used fluid to perform SFE. This has many advantages such as chemically stable, low toxicity, not flammable and inexpensive. Another reason is its low critical temperature (31 °C) and pressure (73.8 bar) for the safe extraction of thermolabile components and its easy separation from the sample during the depressurization step of the process. Supercritical CO_2_ (SC-CO_2_) is a good solvent for the extraction of volatile compounds such as terpenes from *C. sativa* [102,103].

SC-CO_2_ extraction was compared to HD in two studies [80,104]. Both works concluded in a better EO yield in the SFE extract than in the HD extract. The use of SC-CO_2_ has great advantages in comparison to HD, such as the absence of damaged compounds with the use of low temperature, the direct recovery of the EO and the possible fractionation, similar to those done in the works of Da Porto et al. (2014) [104] and Vági et al. (2019) [105], with the isolation of volatile compounds from other extracted components. Moreover, according to [104] SC-CO_2_ extraction would exhibit lower energy consumption compared to hydrodistillation (4.5 kWh/kg compared to 9.6 kWh/kg).

The focus is on the effect of temperature and pressure in all studies of *C. sativa* inflorescence terpene extraction with SC-CO_2_. Da Porto et al. [104] studied the influence of the extraction pressure on the yield and composition of hemp EO. At 40 °C, an increase in extraction pressure from 100 bar to 140 bar leads to a decrease in the EO yield, from 0.67 w% DW to 0.34 w% DW, respectively. In addition, the authors observed that the terpene repartition in the extract at 100 bar is closer to the original inflorescence composition. A lower monoterpene content and higher sesquiterpene content have been noticed in the extract at 140 bar. This different profile is probably induced by a change in the components’ solubility in SC-CO_2_ due to the increase in pressure. According to Naz et al. (2017) [80], an increase in temperature from 40 °C to 45 °C enables a better cannabis EO yield; however, further temperature increase causes a lower yield. The authors also varied the pressure when increasing the temperature, it is thus difficult to attribute this result to the sole temperature factor. Another study used a design of experiment to optimize the SC-CO_2_ extraction of terpenes and cannabinoids from cannabis [106]. The authors claimed that the use of low temperature (35 °C) and no co-solvent favors the extraction of volatile compounds. The influence of extraction pressure is not mentioned. However, the optimal conditions include an extraction pressure of 100 bar which is consistent with the previous cited work [104]. SC-CO_2_ is also used for the recovery of cannabinoids from cannabis, present in the same plant organs than terpenes. Interestingly, a sequential extraction is possible, where the terpenes are extracted without any co-solvent. A second step consisting in the addition of ethanol as co-solvent (20% of CO_2_ flow rate) enables the extraction of cannabinoids [106]. Up to 1.5 g/100 g DW of volatile compounds have also been recovered from cannabis in a separate fraction from cannabinoids. This time, the extract is fractioned in a first separator retrieving the cannabinoids fraction while a second one recovers the terpene fraction [105].

The studies reported here provide interesting perspectives for the SC-CO_2_ extraction of terpenes from *C. sativa*, where low pressure, low temperature and no co-solvent are preferred. Furthermore, the fractionation or sequential extraction are good ways to valorize the entire extract [105,106].

#### 3.2.4. Other Extraction Techniques

The extraction processes also applied for the recovery of terpenes include MAE, UAE and RSLDE. Omar et al. [106] used a design of experiment to obtain the optimal operating conditions during the UAE of cannabis inflorescence. The ultrasound amplitude and solvent composition (mixture of cyclohexane and isopropanol) has a significant influence on the terpenes content while the sonication time has no effect in the studied range. In the optimized conditions, UAE (isopropanol:cyclohexane 1:1, 5 min, 80% amplitude, 3 s^−1^ cycles) where more efficient than SFE, where 80% of the extractable terpenes are recovered in one extraction against 60–70% for SFE with pure CO_2_. SFE were also find to provide lower yields of volatile compounds compared to UAE using MeOH:chloroform (9:1) during 15 min at 40 °C [107]. Ethanol, chloroform or its mixture with ethanol were successfully used without any optimization of the process, for identification purpose [54,77,108]. In all cases UAE is performed in minutes compared to hours in the case of distillation, which should induce a saving of energy.

The MAE of cannabis dry female inflorescence were studied by Fiorini et al. (2020) [109]. The design of experiment included the optimization of the microwave power, time, and biomass moisture (modulated by addition of water). Microwave power and time significantly increased the EO yield while increasing the biomass moisture had the opposite effect. However, the increased EO yield was only correlated to higher contents of cannabinoids. In the optimized conditions (1.05 W/g–113.6 min–28.2% water), the EO yield were similar to HD in a reduced time; however, a different profile was observed: cannabidiol and sesquiterpene contents were favoured over monoterpenes. Indeed, the high energy provided by the microwaves increases the decarboxylation of cannabinoids and causes a loss of the more volatile compounds. In fact, microwaves can also be used as a pre-treatment method to increase cannabinoids and sesquiterpenes contents in the EO [87]. A microwave pre-treatment prior distillation has been compared to a heating pre-treatment with an oven. A short heat treatment in the oven (up to 3 min at 120 °C) or in the microwave (1 min, 450 or 900 W) does not significantly affect the EO yield, whereas prolonged treatments lead to lower yields. Grinding the biomass before the different pre-treatments does not affect the results. All the treatments (oven, microwaves, with or without grinding) led to an overall loss of monoterpenes. However, the smallest decrease was observed when applying microwaves (1 min, 900 W) to unground material. Besides, microwave pre-treatment under these conditions induced the highest contents of sesquiterpenes, mainly caryophyllene (51.6%), α-humulene (6.9%), and caryophyllene oxide (20.5%). The concentrations of these components in the extract of untreated inflorescences were 29, 4.3, and 11.1%, respectively.

RSLDE was successfully employed to recover terpenes from hemp inflorescence. An increase in extraction time caused slight differences in terpene profile [54]. This innovative extraction technique requires no heating and might be interesting for the extraction of the volatile fraction of cannabis.

As terpenes and cannabinoids are present in the same plant organ, they are often co-extracted. A very recent study in 2020 aimed to compare traditional extraction methods and develop new methods of extracting cannabinoids without damaging the terpenes in cannabis [110]. The application of heat pre-treatment is needed for the cannabinoid’s decarboxylation; however, mild temperatures prevent the loss of terpenes. The formulation of products containing both cannabinoids and terpenes is profitable for human health as defined in the entourage effect theory.

In general, novel extraction techniques are still to be investigated for the extraction of cannabis EO. Diverse methods may be optimized, depending on the target compounds.

## 4. Discussion

*Cannabis sativa* L. is a controversy plant due to the psychoactive content of the drug-type cultivars. However, the hemp type gets increased interest due to the numerous bioactive compounds contained in its by-products. Besides the well-known cannabinoids present in cannabis inflorescences, PC and terpenes extracted from hemp deserve particular attention.

After reviewing the available articles on the extraction processes of PC from *C. sativa*, we noted a dominancy of UAE and CSE techniques. This means that even though CSE has several disadvantages such as long extraction time and a use of large amount of solvent [111], it is still frequently used by researchers for its simplicity. Furthermore, due to their low cost, CSE equipment are available in all laboratories. On the other hand, the development of intensified extraction processes such as UAE is under study. This technique is of particular interest due to its numerous benefits including the increase in extraction yield and faster kinetics compared to CSE [58,111]. When optimized, UAE offers the best results in 20 min while extractions often last 3 h in the case of CSE. Other intensified processes such as MAE and ASE have also been used for the extraction of PC from cannabis. Once again, the significant improve lies in the extraction time, reduced to minutes. Not surprisingly, all techniques converge on the use of polar solvents, with aqueous acetone being the most efficient. However, based on the identified studies, further developments are needed for their application on the recovery of PC from hemp. Indeed, many authors have focused on intensifying the process without defining the applied operating conditions. For example, the temperature has a clear influence on PC recovery, but was sometimes not mentioned. The ultrasonic or microwave power shall also be chosen with care knowing the risk of PC degradation. The comparison with and without intensification seems then difficult without defining the objective of PC recovery. The most studied parts of the plant are the hemp seed meal and the inflorescences. These parts are considered as by-products obtained after the extraction of seed oil and fibers. Therefore, research is focused on their valorization by identifying secondary metabolites having biological activities. In this context, PC has interesting potential. Some of the most valuable identified phytochemicals are cannflavins, *N*-*trans-*caffeoyltyramine, and cannabisins. However, stilbenoids presented in Section 2.1 have only been recorded in few PC extracts. They are potentially in a too little amount, need the application of other extraction techniques or have no commercial interest yet. Thus, the development of extraction processes to increase all PC yields is a necessary step and intensified processes such as UAE, MAE and ASE represent great potential for this objective.

Cannabis essential oil exhibited numerous properties, and thus attracted researcher’s attention in the late years. Most studies using solvent extraction date from before 2011. The majority of recent studies (from 2018) use greener extraction processes such as SD and HD. Additionally, even though the application of SC-CO_2_ to EO extraction is still scarce in cannabis literature, it appears that scientists are beginning to apply these intensified process in their research. After reviewing the operating conditions of the hydrodistillation process used in the different studies, the conclusions are as follows: (1) liquid/solid ratio between 6 and 35 mL/g, (2) extraction time between 2 and 4 h, and (3) temperature between 110 and 130 °C. The recovery of terpenes from *C. sativa* EO varies deeply with the distillation technique and the pre-treatment applied to the biomass. The highest yield is obtained from fresh cannabis and HD process without pre-treatment. Regarding the EO composition, a loss of the most volatile components, mainly monoterpenes, is observed during HD in comparison with SD, or after pre-treatment of the biomass (e.g., drying, application of microwaves). On the contrary, increased contents of bioactive sesquiterpenes and cannabinoids are obtained, which seems very interesting for further valorization. The SC-CO_2_ extraction process allowed to obtain a higher quality of EO with an energy consumption divided by 2 compared to the HD process. The other benefit of SC-CO_2_ is the low operating temperature, with an optimum recovery at 100 bar. Several studies mention the co-extraction of terpenes and cannabinoids. Depending on the operating conditions and no matter the technique, cannabinoids are recovered in cannabis EO and terpenes are also extracted when the focus is on cannabinoids. This may be of interest, knowing the interactions between these two classes of metabolites. When a separate collection is needed, it is possible to partition the extraction using either HD or SC-CO_2_. A remarkable point in the extraction of EO from *C. sativa* is the recovery of a solvent-free extract when CO_2_ is used without any co-solvent. Additionally, a method close to solvent-free microwave extraction was successfully used, offering an extraction yield similar to HD in a shorter time. The extraction of triterpenes from *C. sativa* have only been conducted with CSE in polar solvents and require further development.

## 5. Conclusions

Different processes including common and emerging techniques have been used to recover phenolic compounds and terpenes from cannabis. Considering the need to develop sustainable processes, it seems important to explore eco-friendly solvent extraction techniques or solvent-free methods that will combine high yield and safe recovery without damage or loss of the target compounds. In the same way, the use of intensification and fractionation techniques such as UAE, MAE, RSLDE or SC-CO_2_ are good solutions to increase the extraction’s efficiency in a shorter time. Only a combination of these methods will enable the valorization of all compounds having biological activities from by-products of *C. sativa* in a biorefinery approach.

## Figures and Tables

**Table 1:** Studies using conventional solvent extraction for the recovery of PC from cannabis.

Plant Variety	Plant Organs	Solvent	Operating Conditions	Best PC Results	Best Biological Activities	Ref.
Futura 75	Seeds	EtOH 80%	10 mL/g, 4 °C, 3 h	TPC 2.33 mg GAE/g DW of extract TFC 2.93 mg QE/g DW of extract	ORAC assay: 134 μmol TE/g DW seeds 40% DPPH inhibition in seeds extract	[3]
Futura 75	Seeds	MeOH 80%	5 mL/g, 3 h	TPC up to 80.4 mg GAE/g of extract TFC up to 92.8 mg RE/g of extract	IC 50 (DPPH) = 0.15 mg/mL	[48]
Carmagnola	Seeds	MeOH 80%	5 mL/g, 3 h	TPC up to 67.0 mg GAE/g of extract TFC up to 97.8 mg RE/g of extract	IC 50 (DPPH) = 0.17 mg/mL	
Felina 32	Seeds	MeOH 80%	5 mL/g, 3 h	TPC up to 72.7 mg GAE/g of extract TFC up to 109.0 mg RE/g of extract	IC 50 (DPPH) = 0.10 mg/mL	
Helena	Aerial parts	Water EtOH 30, 50, 70, 90%	20 mL/g, RT, 24 h	Best solvent: EtOH 50% TPC up to 17.05 mg GAE/g DW TFC up to 11.20 mg CE/g DW	Best DPPH EC50 activity = 50% EthOH (0.1331 mg hemp DW/mL) Best RC EC50 activity = 30 and 50% EtOH (0.4450 and 0.5327 mg hemp DW/mL)	[49]
Nd	Seed meal	MeOH 80, 100%; EtOH 100%; Acetone 80, 100% MAW (35:35:30 *v*/*v*/*v*)	16.6 mL/g, 25 °C, 1 h	Best solvent: MAW (TPC 7.33 mg GAE/g FW; TFC 0.27 mg LUE/g FW) Caffeic acid up to 8.31 mg/100 g FW in MeOH extract Quercetin up to 104.11 mg/100 g FW in MAW extract Luteolin up to 46.11 mg/100 g FW in MAW extract	DPPH: 16.79% DPPH inhibition in MAW extract FRAP: 3.51 μmol Fe (II)/g FW in MAW extract	[50]
Nd	Leaves	Acetone 50, 100% MeOH 50, 100%	10 mL/g, RT, 2, 8 or 18 h	Increase in TPC with extraction time Best conditions: MeOH, 18 h (TPC 0.890 mg GAE/g)	Increase in reducing power with extraction time MeOH, 18 h: Absorbance = 0.866 Antimicrobial activity against *S. aureus*, *C. albicans* and *P. aeruginosa*	[51]
Helena	Seed meal	CD/water mixtures (1–40%)	5–15.2 mL/g, 20–60 °C, 3 h	Optimum operating conditions: 32.1% of CD, 15.2 mL/g at 28 °C TPC 4.5 mg/g DW	DPPH: 12.4 µmol TRE/g DW in optimized conditions (32.1% of CD, 15.2 mL/g, 28 °C)	[52]
Nd	Leaves and flowers	MeOH	30 mL/g, 40 min	Canniprene up to 2.09 mg/g DW Cannflavin A up to 0.28 mg/g DW, cannflavin B up to 0.11 mg/g DW	Anti-inflammatory activity of canniprene	[34]
Nd	Leaves	EtOH, MeOH, ethyl acetate, CHCl_3_, acetone, hexane, water	4 mL/g, RT, 72 h	Best solvent: MeOH (TPC 36.42 mg GAE/g extract; TFC 59.03 mg QE/g extract)	55.57% DPPH inhibition in acetone 54.51% DPPH inhibition in ethanol 49.50% DPPH inhibition in methanol	[53]
Futura 75	Inflorescence	EtOH	5 mL/g, RT, 30 days	TPC 125.12 mg GAE/g extract Gallic acid 36.02 µg/g extract *p*-OH-benzoic acid 52.41 µg/g extract Ferulic acid 96.70 µg/g extract Rosmarinic acid 328.21 µg/g extract Luteolin 127.67 µg/g extract	FRAP: 83.14 mg TE/g extract DPPH: 32.43 mg TE/g extract ABTS: 502.16 mg TE/g extract	[54]
			5 mL/g, 100 °C, 2–6 h	TPC up to 124.25 mg GAE/g extract Gallic acid up to 408.92 µg/g extract *p*-OH-benzoic acid up to 95.18 µg/g extract Caffeic acid up to 36.98 µg/g extract Rosmarinic acid up to 206.30 µg/g extract Luteolin up to 753.01 µg/g extract Apigenin up to 54.22 µg/g extract	FRAP: 78.69 mg TE/g extract DPPH: 31.54 mg TE/g extract ABTS: 433.22 mg TE/g extract	

ABTS: 2,2′-azino-bis (3-ethylbenzothiazoline-6-sulphonic acid). RC: reductive capacity of ion Fe^2+^ into Fe^3+^. CD: aqueous solution of 2-hydroxypropyl-β-cyclodextrin. CE: catechin equivalent. DPPH: 2,2-diphenyl-1-picrylhydrazyl or 1,1-diphenyl-2-picrylhydrazyl. ORAC: oxygen radical absorbance capacity. DW: dry weight. EC50/IC50: concentration of extract allowing 50% of inhibition/ion reduction. FRAP: ferric reducing antioxidant power. FW: fresh weight. GAE: gallic acid equivalent. LUE: luteolin equivalent. MAW: methanol, acetone, water. Nd: not defined. QE: quercetin equivalent. RE: rutin equivalent. RT: room temperature. TE/TRE: trolox equivalent. TFC: total flavonoid compounds. TPC: total phenolic compounds.
